# THE EXPERIENCES OF DISCLOSING ALZHEIMER’S DISEASE IN THE US WORKPLACE

**DOI:** 10.1093/geroni/igae098.3575

**Published:** 2024-12-31

**Authors:** Allison Gibson, Kathryn Coccia, Lauren Hasten

**Affiliations:** Saint Louis University, St Louis, Missouri, United States; Saint Louis University, Chicago, Illinois, United States; Saint Louis University, St. Louis, Missouri, United States

## Abstract

With the shift of aging adults delaying retirement, more individuals are being diagnosed with Alzheimer’s disease and related dementia (ADRD) while still in the workforce. Using a modified grounded theory approach, 16 people living with ADRD were interviewed about their experience receiving a diagnosis while still in full-time employment (mean age 66). Thematic analysis was employed to examine the study’s aims with member checking and an audit trail to enhance study credibility and trustworthiness. Many study participants shared their diagnosis with employers, resulting in positive experiences. Study findings indicated that participants were more likely to disclose their diagnosis to their employer when they felt supported by their supervisor, perceived their employer would offer them reasonable accommodations to continue working, or felt they were well positioned to retire. Those who did not disclose their diagnosis did so because they feared reasonable accommodations were either not available or accessible, and they reported significant financial stress. Study implications suggest those newly diagnosed need support regarding ADRD and employment, and human resources personnel need more training on ADRD workers. While not all participants were able to continue in the workforce following diagnosis, many could through flexible work roles and supportive environments. Many study participants were able to continue in their full-time positions until they retired or elected to shift employment to a new role or employer. However, some experiences highlight the continued challenges of accessing disability resources. This presentation will conclude with recommendations for specialized policies, training, and future research.

